# Psychometric Evaluation of a Consumer-Developed Family-Centered Care Assessment Tool

**DOI:** 10.1007/s10995-015-1709-y

**Published:** 2015-04-08

**Authors:** Nora Wells, Suzanne Bronheim, Stephen Zyzanski, Clarissa Hoover

**Affiliations:** 1 Family Voices, Inc., Albuquerque, NM USA; 2 Georgetown University, Washington, DC USA; 3 Case Western Reserve University, Cleveland, OH USA

**Keywords:** Family-centered care, Quality improvement, Patient engagement, Patient satisfaction, Patient experience

## Abstract

The objective of this study was to create a psychometrically sound measure of family-centered care, the Family-Centered Care Assessment (FCCA), developed through a process led by families in collaboration with maternal and child health leaders. The items for the FCCA scale were initially developed by families of children and youth with special needs in partnership with pediatric providers and researchers. Using an Institutional Review Board-approved research protocol, the questions were revised based on input from focus groups of diverse parents in three states. Parental responses (N = 790) to the revised 59-item survey were collected online from families in 49 states. Item distributions uniformly showed excellent spread. A principal axes factor analysis confirmed the existence of a single factor. Rasch modeling item analyses identified a reduced subset of 24 items that demonstrated excellent psychometric properties. All items met the criteria for a linear Rasch scale. Empirical evidence in support of the construct validity of the 24-item measure was derived: all items had a positive and substantial item–total correlation; person alpha scale reliability was >0.80 and the item reliability was >0.90; both separation indices were >2.0; infit and outfit statistics were within 0.5–1.5; and item difficulties ranged between −2 and +2 logits. Strong rank-ordered associations and large effect sizes were observed for six indicators of quality of care. This study’s family-led process produced a tool, the FCCA, to measure families’ experience of care with excellent psychometric properties.

## Introduction

The concept of family-centered care has been central in health services for the estimated 14.6 million [1] children and youth with special health care needs (CYSHCN) and their families for over 20 years, guided by the legislative mandate of the 1989 Omnibus Budget Reconciliation Act and the federal Maternal and Child Health Bureau (MCHB) [2].

Family-centered care is a way of providing services that assures the health and well-being of children and their families through respectful family–professional partnerships. It honors the strengths, cultures, traditions, and expertise that families and professionals bring to this relationship. Family-centered care is a standard of practice which results in high quality services [3]. Partnerships between families and professionals are built on the following principles [4]:

- Families and professionals work together in the best interests of the child and the family.
- As the child grows, s/he assumes a partnership role.
- Everyone respects the skills and expertise brought to the relationship.
- Trust is acknowledged as fundamental.
- Communication and information sharing are open and objective.
- Participants make decisions together.
- Families and professionals share a willingness to negotiate.

Based on this partnership, family-centered care [5, 6]:

- Acknowledges the family as the constant in a child’s life.
- Builds on family strengths.
- Supports the child in learning about and participating in his/her care and decision-making.
- Honors cultural diversity and family traditions.
- Recognizes the importance of community-based services.
- Promotes an individual and developmental approach.
- Encourages family-to-family and peer support.
- Supports youth as they transition to adulthood.
- Develops policies, practices, and systems that are family-friendly and family-centered in all settings.
- Celebrates successes.

Family-centered care is reported to improve the patient’s and family’s experience with health care, reduce stress, improve communication, reduce conflict (including lawsuits), and improve the health of children with chronic health conditions [7, 8]. Patient- and family-centered care is endorsed by the Institutes of Medicine [9], American Academy of Pediatrics (AAP) [7], and the US Department of Health and Human Services [10] and is designated as one of the core components of a medical home by the AAP [11].

Six core outcomes related to services and supports for CYSHCN [12] and their families in the United States have been set forth by MCHB and nationally recognized as essential components of a well-functioning system of services, including one that “families of children and youth with special health care needs (CYSHCN) partner in decision making at all levels and are satisfied with the services they receive.” [13, 14]. In clinical settings, this outcome takes the form of family-centered care [13, 14].

Given the centrality of family-centered care within the field of Maternal and Child Health, an instrument to measure family-centered care that is psychometrically sound and validated, and that reflects both families’ and professionals’ perspectives is critically important. A measure of family-centered care should include items that reflect the full range of principles and components that have been deemed critical to families. Table 1 lists the foundations and components of family-centered care and the necessary areas for measurement in a tool that is inclusive of the family’s perspective of the concept. A reliable process for developing measurement tools also depends on effective family professional partnerships.

**Table 1:** Topical areas for measurement of the foundations and components of family-centered care

Topical areas for measurement	Foundations and components of family-centered care
Communication with providers	Communication and information sharing are open and objective
Decision-making interactions with providers	Trust is fundamental
	Participants make decisions together
	Families and professionals share a willingness to negotiate
Future orientation–planning, promotion, and prevention	As the child grows, s/he assumes a partnership role
	Supports the child in learning about and participating in his/her care and decision making
	Promotes an individual and developmental approach
	Supports youth as they transition to adulthood
Strengths-based approach to care	Mutual respect for the skills and expertise each partner brings to the relationship
	Builds on family strengths
Care coordination to lessen family burden of care	Recognizes the importance of community-based services
Cultural and linguistic competence in care	Honors cultural diversity and family traditions
Practice structure, function, and policies to address family-centered care	Develops policies, practices, and systems that are family friendly and family-centered in all settings
Family support and capacity building	Acknowledges the family as the constant in a child’s life
	Encourages family-to-family and peer support

Existing tools are available to measure aspects of family-centered care, but each has limitations. Some are intended only for use in inpatient settings [15]; others assess family-centered care at the level of the health care organization, or provide only a high-level report on families’ overall experience in the care setting [16, 17] rather than on their interactions with a specific health care provider. Although many instrument developers have included family members in the development of their tool [18], no tools have been created under the leadership of families. One promising tool, the Family-Centered Behavior Scale [19], was developed with extensive family input, but it has not been used in subsequent published research or quality improvement efforts.

The most widely used measures of family-centered care are the Medical Home Family Index (MHFI) [18] and the Consumer Assessment of Healthcare Providers and Systems (CAHPS) Clinician and Group Survey: 12 month Survey, Child [20]. Both had input from families in their development. The CAHPS measure underwent both cognitive and psychometric testing [20] and the MHFI has demonstrated the ability to discriminate changes in family experience within the context of medical home improvement [18]. However, neither instrument included items addressing the full range of themes that capture the family perspective on family-centered care. Table 2 illustrates the gaps in these two measures, in particular regarding decision-making interactions with health care providers and cultural and linguistic competence. Thus, there continues to be a need for a psychometrically sound, validated measure of family-centered care that reflects the full range of concepts that families deem essential.

**Table 2:** Topical areas of family-centered care as measured by CAHPS, MHFI, and FCCA

Topical areas for measurement	CAHPS clinician and group survey: 12-month survey, child questions (item numbers)	Medical home family index questions (item numbers)	Family-centered care assessment
Communication with providers	5–11, 21, 22	2, 3, 6	1
Decision-making interactions with providers			2–5
Future orientation–planning, promotion, and prevention	29–41	18	6–8, 24
Strengths-based approach to care		4	11, 12
Care coordination to lessen family burden of care		11, 19	19
Cultural and linguistic competence in care			16–18
Practice structure, function, and policies to address family-centered care	2, 42, 43	1, 2, 7–10, 21–23	9, 10, 22, 23
Family support and capacity building		5, 12, 13, 15, 20	13–15, 20, 21

This paper describes the processes by which a measure of family-centered care has been developed with families as leaders on the research team. Families worked closely with professionals to identify the concepts of family-centered care to be included in the construction of this measure.

## Methods

In two meetings in 2007, Family Voices, the AAP, and the MCHB convened 22 family leaders, pediatric practitioners, and academic pediatricians who had extensive experience with family-centered care. Participants identified the need for a set of indicators that would help to guide implementation of family-centered care in the field. Family Voices, a national, family-led family advocacy organization that promotes quality of care for families of CYSHCN, took the lead in this task. Using in-person meetings, interviews, and conference calls, two self-assessment tools were drafted, one each for families and health care providers. Questionnaires included 98 questions in the Family-Centered Care Self-Assessment Tool for Families and 105 questions in the Family-Centered Care Self-Assessment Tool for Providers. The self-assessment questions were grounded in the concepts that had previously been identified by families of CYSHCN as important in quality of care, and were critically reviewed by expert pediatric providers and policymakers from the AAP, MCHB, schools of public health, and by researchers.

The two self-assessment tools were first tested through individual surveys completed by pediatricians and families of CYSHCN in Pennsylvania and in Massachusetts. Almost all of the families and pediatricians who provided this feedback indicated that they would recommend the use of the tools for setting expectations and/or for quality improvement discussions. A number of investigators and clinicians expressed interest in using the tools. However, respondents expressed consensus that the tools would have to be substantially shortened to be most useful.

This article reports on the subsequent reduction and validation of the tool that had been developed for families. In the fall of 2011, a team of three Family Voices expert family leaders and two university research faculty was assembled to implement the psychometric evaluation. A research protocol was established and all work was completed under the supervision of the Western Institutional Review Board. Each investigator completed subject protections training through the Collaborative Institutional Training Initiative.

Through expert review, the number of items in the instrument was reduced to 68 and the language simplified. The shortened instrument was subjected to testing in a series of focus groups in three states with a total of 36 parents and other caregivers of CYSHCN, the majority of whom were Hispanic and/or nonwhite (Table 3). Participants were asked to complete the questionnaire and then discuss items that they could not answer or found confusing. Following the focus groups, 59 questions were selected and revised based on the focus group findings.

**Table 3:** Focus group participants

*Location*	
New Jersey	16 (2 groups)
New Mexico	3
California	17 (2 groups)
*Child’s diagnosis*	
Emotional/behavioral	9
Developmental	16
Chronic illness	8
*Age of child*	
Under 3 years	3
3–14 years	24
14–21 years	7
Not specified	2
*Primary insurance*	
Public	18
Private	18
*Race*/*ethnicity*	
Non-hispanic white	14
Hispanic and/or nonwhite	22

The 59 questions, written at an eighth-grade reading level, were formatted into an online survey. The online survey also collected demographic information. A series of questions about satisfaction with and trust in the provider were included to help assess the validity of the family-centered care questions. Survey participants were recruited by the national Family Voices organization and by the Family Voices network of family-led organizations in every state using electronic mailing lists, social media, and personal requests. The survey was available for online completion for a period of nine weeks from November 2012 to January 2013. Participants were instructed to respond to the survey based on their experience with only one child (in case the family had multiple CYSHCN) and one health care provider.

Demographic characteristics of the Family-Centered Care Assessment (FCCA) survey respondents are given in Table 4. Compared to respondents in the nationally representative National Survey of Children with Special Health Care Needs (NS-CSHCN), FCCA participants were more likely to be white, non-Hispanic, and somewhat more likely to have private insurance. Their children were considerably more likely to meet multiple criteria of the NS-CSHCN screener, indicating greater severity of health disability [21].

**Table 4:** Demographic and health care setting characteristics of online survey respondents

Characteristics	N	%
Health care setting		
Care setting		
Private office	549	69
Hospital clinic	169	21
Community clinic	45	6
Other	31	4
Health care provider		
Physician	653	82
Nurse practitioner/physician’s assistant	23	3
Other	119	15
Medical home practice		
Yes	118	15
No	567	71
Not sure	11	14
Respondent		
Relation to child		
Parent	755	95
Grandparent/other relative	31	4
Other	8	1
Geographic location		
Rural	234	29
Urban	187	24
Suburban	375	47
Gender		
Female	739	94
Male	48	6
Child		
Race		
White	660	83
African American	50	6
Other	84	11
Ethnicity		
Non-hispanic	724	92
Hispanic	66	8
Type of insurance		
Private	559	70
Public	228	29
None	9	1
Special health care needs (all that apply)		
Prescription medication	596	75
More than usual health care or educational services	662	83
Limited in ability	570	72
Therapy services	538	68
Counseling	369	46
None identified	3	1

### Psychometric Analysis

A multistep data analysis process, including exploratory factor analysis, Rasch modeling [22], and differential item functioning (DIF) [23], was used in the development of the FCCA scale. A principal axes factor analysis with varimax rotation was computed on the 59 items to confirm that a single factor accounted for the interitem correlations. The retention of a single factor was based on an examination of the scree plot, the presence of a single large Eigen value, and the number of items with loadings >0.4 defining the factor.

A series of item deletions was carried out using the following criteria: items with low factor loadings (<0.4), low item–total correlations (<0.3), and Rasch misfit statistics (infit and outfit values outside the range of 0.5–1.5). At each step in the analysis, misfit items were removed and the Rasch analysis rerun. The process was repeated until the results showed all remaining items exhibited good Rasch model fit.

Finally, a series of DIF analyses was performed on all items that met the Rasch goodness-of-fit criteria. In Rasch modeling, DIF implies that item difficulty is different for different groups. Such items may be biased toward certain subgroups, which in turn could threaten the validity of the measure and produce misleading results [23, 24]. In this study, DIF attributable to race, ethnicity, gender, and insurance status was assessed. Items with moderate-to-large DIF (DIF size >0.43 logits) [25] were deleted from the final version of the FCCA scale.

### Reliability of the Final FCCA Scale

Rasch person and item reliability statistics [26] were used to evaluate the internal consistency of the final FCCA scale. The person reliability statistic is equivalent to the traditional Cronbach’s alpha [25, 26]. Item reliability, with no traditional equivalent, depicts the level of confidence that items would have the same respective order in another sample of participants. Person reliability statistics of >0.8 and item reliability statistics of >0.9 represent target guideline reliability for both. Rasch analyses also provide a separation index for both persons and items. A high person separation index indicates a wide range of family-centered care scores within the sample studied. A high item separation index indicates that the items cover a useful range of item difficulty appropriate for measuring persons with a wide range of family-centered care scores [27].

### Validity of the Final FCCA Scale

Validity was examined using multiple sources of information. Initially, content validity was established through the use of focus groups and an expert panel as described above. Next, a principal factor analysis was performed to confirm the existence of a single factor. Rasch analysis was then conducted on the single factor items. In Rasch modeling, good item fit statistics and a good match between item difficulty and person ability provide evidence of construct validity [28, 29].

Fit statistics generated by Rasch analysis are used to determine the quality of items. In Rasch model expectations, individuals who perceive lower levels of family-centered care obtain lower scores while those with higher perceived levels have higher scores on any item [30–32]. When all items in a measure are a good fit, this fit provides evidence for the construct validity of the measure [28, 29]. Finally, associations between selected parental indicators of quality of care and FCCA scale scores were computed as further evidence in support of the scales’ construct validity.

Rasch and DIF analyses were computed using the Winsteps 3.75 software [25] and both were based on the Rasch partial credit model [33]. Item difficulty estimates, goodness-of-fit statistics, and item–total correlations were reported. Descriptive and inferential statistics were computed to determine whether FCCA scores differed by gender, age, race, ethnicity, practice setting, geographic location and insurance status of parents, and by the child’s age and years of care.

## Results

### Sample Characteristics

The 796 respondents (Table 4) represent families from 49 states and the District of Columbia. The majority were white (83 %), non-Hispanic (92 %), female (94 %), and parents (95 %). Care was provided in private office settings (69 %) by physicians (82 %) in suburban locations (47 %). Seventy percent of respondents reported that their children had private insurance and the majority required prescription medications (75 %), above average usage of services (83 %), including special therapy (68 %) and counseling (46 %) services, and had functional limitations (72 %). The average child was born in the year 2000, which indicates that family members had, on average, 12 years of experience in caregiving; 65 % of the children had special health care needs before the age of 1 year.

### Item Reduction

Missing item responses were infrequent (<1 %) and imputed by mean substitution. A principal axes factor analysis supported the presence of a single factor (Eigen value = 28.2), accounting for 47 % of the total variance. Items defining this factor focus on important relationships between family caregivers and their health care providers with an emphasis on the nature and extent of family-centered care.

Initially, two items were deleted because of low factor loadings. Two more items were deleted because of poor Rasch item fit statistics (i.e., infit and outfit values out of the range 0.5–1.5) [22] and subsequent DIF analysis for race revealed three items exhibiting significant DIF (>0.43 logits), which indicated potentially biased items. Additionally, six respondents were removed from the analysis because of inconsistent response patterns as detected by Rasch goodness-of-fit statistics. The final sample size for all analyses was 790. Although the remaining 52 items met the criteria for Rasch modeling, they were subjected to an expert panel consisting of family leaders, health care professionals, and researchers, for further item reduction. Items were chosen to reduce redundant items at each level of difficulty and to assure that all topical areas for measurement based on the principles of family-centered care were included, leaving a total of 24 items with good item–model fit and no presence of DIF (Table 5). The estimated item difficulties for the 24 retained items ranged from −2.23 logits (least difficult) to +1.76 logits (most difficult); a range of nearly two standard deviations above and below the mean item difficulty level of 0.0 (Table 5).

**Table 5:** Estimates of item difficulty, standard error (se), mean-square fit statistics and item–total correlations and topical area addressed for items of the FCCA scale

Item	Abbreviated item content: my health care provider …	Item*	SE	Infit	Outfit	ITC	Topical Area+
16	Asks if other community members involved in decision-making	1.76	0.05	1.72	1.69	0.52	CLC
9	Offers other ways that care can be provided	1.42	0.04	1.46	1.32	0.62	PS
18	Asks if alternative healing treatments are to be used	1.27	0.04	1.32	1.28	0.62	CLC
20	Has ways to connect with other families	1.24	0.04	1.01	0.9	0.71	FS
21	Has information to help others understand my child’s needs	1.12	0.04	0.84	0.83	0.74	FS
19	Has ways to help make first contact with community services	0.88	0.04	1	0.96	0.72	CC
15	Asks about the well-being of my whole family	0.74	0.04	0.8	0.75	0.77	FS
14	Asks about emotional stresses in caring for my child	0.66	0.04	0.73	0.68	0.78	FS
17	Asks about family beliefs when developing treatment plans	0.65	0.04	1.1	1.08	0.69	CLC
22	Discusses ways to help pay when insurance does not cover	0.48	0.04	1.09	1.36	0.67	FS
5	Discusses how health care decisions will affect whole family	0.29	0.04	1.12	1.27	0.67	DM
8	Has ways to help child understand treatment before it’s done	0.13	0.04	1.2	1.22	0.66	FPP
10	Has ways to consider my schedule in making appointments	0.05	0.04	1.46	1.52	0.61	PS
24	Asks about what I hope for my child’s future	−0.17	0.04	0.73	0.7	0.76	FPP
23	Has ways to help our understanding of the medical record	−0.36	0.04	0.98	1.02	0.66	PS
13	Helps me to change my child’s treatment plan when needed	−0.44	0.04	0.85	0.9	0.7	FS
6	Helps me plan for big changes in my child’s life	−0.67	0.04	0.82	0.76	0.72	FPP
11	Asks me what is working well in my child’s health care	−0.80	0.04	0.79	0.7	0.72	SB
7	Discusses my child’s overall health and well-being	−0.91	0.04	0.93	0.89	0.66	FPP
12	Recognizes my strengths in caring for my child	−1.12	0.04	1.07	0.87	0.65	SB
3	Decide together on goals for my child’s treatment	−1.17	0.04	0.79	0.74	0.68	DM
4	I’m comfortable disagreeing with care recommendations	−1.35	0.05	1.05	1.11	0.54	DM
2	Supports the role I want to take in my child’s care	−1.46	0.05	0.72	0.74	0.64	DM
1	Discusses my child’s care in words I understand	−2.23	0.06	1.04	1.29	0.44	CM

* Item = item logit score; SE = standard error of item score; infit = mean square for redundancy; outfit = mean square for outliers; ITC = item–total correlation

*+CLC* cultural and linguistic competence, *PS* practice structure, *FS* family support, *CC* care coordination, *DM* decision making, *FPP* future/promotion/prevention, *SB* strengths-based, *CM* communication

### Scoring

FCCA scores were computed by summing the 24 individual items. Each item was rated on a 5-point Likert scale (1 = almost never; 5 = almost always). FCCA scores have a maximum range of 24–120, with high scores indicating greater perception of family-centered care. A neutral score is represented by a score of 72. The study mean of 76.2 (standard error 0.75) indicates a positive perception on average.

### Reliability

The Rasch person reliability coefficient was 0.95, which indicates high internal consistency reliability. The Rasch item separation index was 23.5 and is considerably above the minimum index score of 2.0 [25]. FCCA items also demonstrated excellent internal consistency with an item reliability of 1.00.

### Construct Validity

**Fig. 1:**
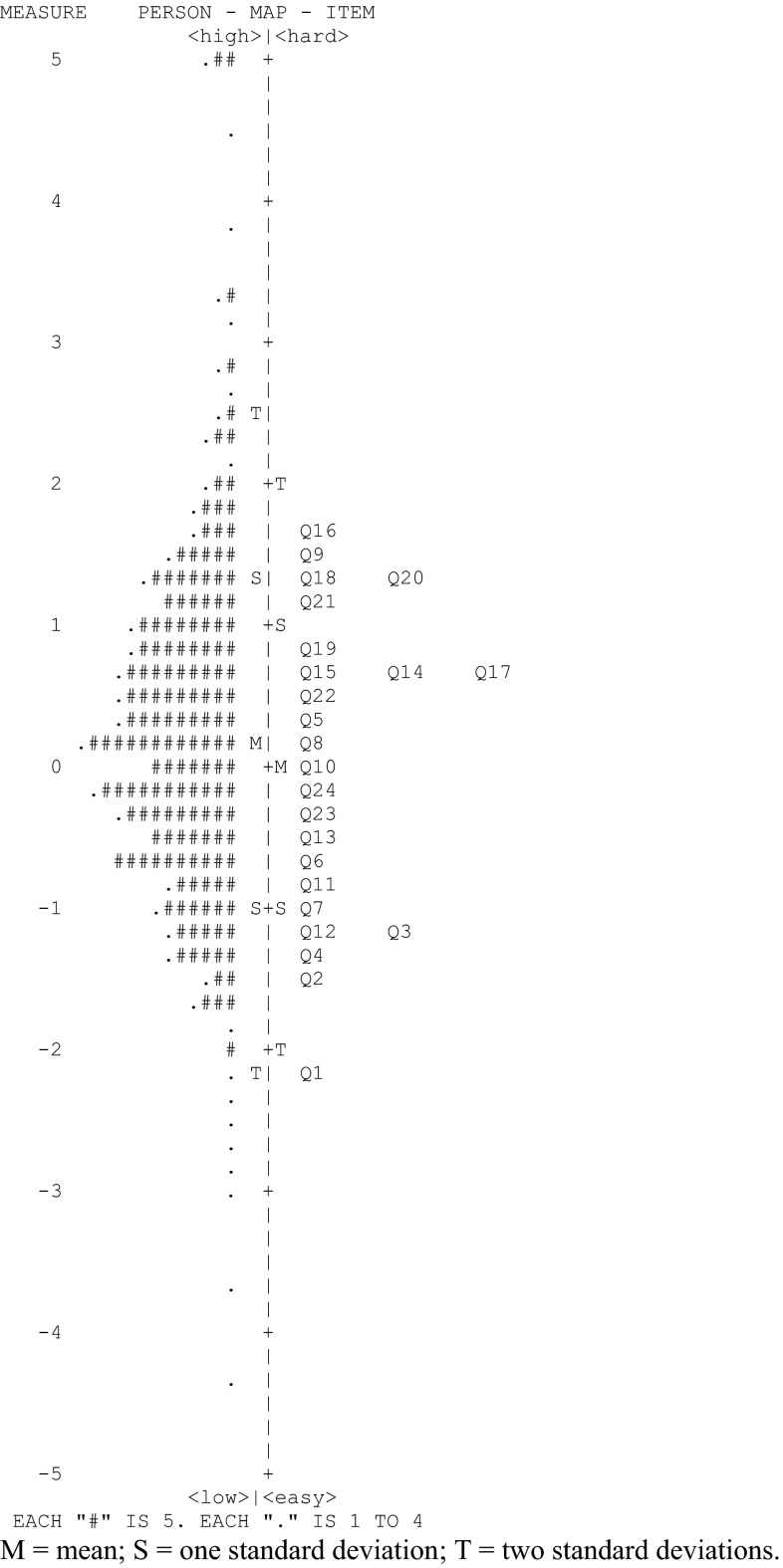
Item–person map of the 24 items comprising the FCCA scale. *M* mean, *S* one standard deviation; *T* two standard deviations. Table 5 provides abbreviated item content for each question

According to Rasch fit statistics, all 24 items of the FCCA fitted (Table 5), which indicates that a single factor model is appropriate. A principal axes factor analysis of the original 59-item pool revealed one dominant factor (Eigen value = 28.2), which adds further evidence that the majority of items measure a similar construct. Moreover, since the FCCA includes no DIF items, scale scores hold measurement invariance across different demographic subgroups, which supports the validity of the FCCA further. A comparison between item difficulty level and the family’s perception of care is illustrated by the item–person map of Fig. 1 and provides further evidence of the construct validity of the FCCA. The item–person map displays the location and distribution of both items and family care perceptions on the same common logit metric. In Fig. 1, a numeric logit scale is the left column, family scores are charted in the middle column, and items are charted on the right. Items at one level of difficulty are distinct from items at another level. Families with higher family-centered care scores and the more difficult items to endorse are to be found at the top of the map.

The mean item difficulty level is standardized at 0.0, while the mean person difficulty level was observed to be 0.25, indicating that, on average, the items were somewhat easier to endorse and that respondents had a slightly higher family-centered care orientation than that of the items. However, the closeness of the item and person means indicates that the respondents were well assessed by the items. Item difficulties ranged from −2.23 to 1.76 logits while respondent family-centered care scores ranged from −4.33 to 5.69 logits. Eight percent of the sample had scores on the high end that were outside the observed maximum item difficulty level, which suggests that additional items are needed to assess persons with especially high levels of family-centered care.

### Demographic Differences

In analyzing mean FCCA scores by respondent demographics, statistically significant differences in scores were noted only for gender (Table 6). Male respondents had higher mean scores (83.9) than female respondents (76.0) with *P* = 0.012 and a moderate-to-small effect size of 0.38. Neither the child’s age (r = –0.05, *P* = 0.19) nor years of care (r = –0.03, *P* = 0.37) were associated with FCCA scores.

**Table 6:** Association of FCCA scale scores with characteristics of family caregivers

Variable	N	Mean	t/ANOVA	*P* value	Effect size
*Ethnicity*					
Non-hispanic	720	76.7	2.2	0.628	0.29
Hispanic	64	70.7			
*Gender*					
Male	47	83.9	2.52	0.012	0.38
Female	734	76			
*Relationship*					
Parent	750	75.9	0.98	0.337	0.23
Grandparent/relative	26	80.8			
*Insurance*					
Private	554	76.4	0.71	0.931	0.02
Public	227	76			
None	9	74.1			
*Race*					
Caucasian	657	76.1	0.77	0.461	0.18
African American	49	79.8			
Other	82	75.5			
*Setting*					
Private office	545	76.5	0.53	0.66	0.03
Hospital clinic	168	77.1			
Health center	45	74.6			
*Place of residence*					
Rural	232	77.7	1.35	0.26	0.16
Urban	184	74.3			
Suburban	374	76.3			

### Concurrent Validity

Strong rank-ordered associations and large effect sizes (>0.80) were observed for six indicators of quality of care (Table 7). These indicators included recommending the health care provider to other families with similar children, no interest in changing health care providers, feeling like partners in the child’s health care, trust in the health care provider’s judgments about the child’s care, satisfaction with the care received from this provider, and the health care provider’s practice being described as a medical home.

**Table 7:** Association of FCCA scale scores with family caregiver perceptions of care

Perceptions	N	Mean	F-test	*P* value	Effect size
*Recommended provider*					
SD/D/N*	202	59	224.84	0.0001	1.85
Agree	264	72.3			
Strongly agree	324	90.2			
*Change provider*					
Strongly disagree	243	90.2	81.32	0.0001	1.67
Disagree	240	77.4			
Neutral	144	69.9			
Agree	98	61.7			
Strongly agree	62	55.1			
*Feel like partner*					
Almost never	22	41.1	168.4	0.0001	2.25
Rarely	48	49.8			
Sometimes	114	58.2			
Usually	197	72.3			
Almost always	404	88.4			
*Trust in provider*					
Strongly disagree	19	39.8	179.92	0.0001	2.31
Disagree	28	45.5			
Neutral	108	57.5			
Agree	309	72.3			
Strongly agree	324	91.1			
*Satisfied with care*					
Strongly disagree	24	42.9	187.39	0.0001	2.07
Disagree	50	51.5			
Neutral	110	58			
Agree	262	72.6			
Strongly agree	340	90.9			
*Medical home*					
Yes	115	88	23.81	0.0001	0.68
No	565	73.6			
Not sure	110	77.4			

* Strongly disagree, disagree, and neutral were combined because of the similarity of means

## Discussion

This study has resulted in a highly reliable scale of 24 items to measure family perceptions of the family-centeredness of child health care from a provider. Study data provided evidence of the scale’s content, construct, and concurrent validity. The scale’s content validity is based on the knowledge and recommendations of national experts in family-centered care, who engaged in an intensive iterative process, including literature review, discussions, focus groups, and pilot testing to develop the initial instrument item pool. Construct validity was supported by all items in the FCCA scale having a good fit under the Rasch model, being DIF free, and having item–total correlations that were positive and close to their expected values. Concurrent validity was documented by significant associations of FCCA scale scores with other important indicators of quality of care as reported in Table 7. Responses to subjects’ ratings showed sufficient variability to allow for effective analysis. Response patterns on key questions such as “feel like a partner” and “satisfied with care” were comparable to responses reported elsewhere in the literature [34, 35]. These associations, with their large effect sizes, demonstrate both statistical and clinical significance [36]. The lack of difference in scale scores by all demographic variables except gender indicates that the scale can provide an accurate assessment of family-centered care with various demographic subgroups. The item–person map indicates good item difficulty and person ability match, which suggests that the family-centered care scores for the majority of the respondents were well assessed by the 24 items of this scale.

The extensive process for gathering family input in development of the items tested resulted in a set of items that were understandable and pertinent to families as demonstrated in the very low missing item rate (<1 %) and few items excluded due to low factor loading, poor Rasch item fit statistics, or DIF. This process expanded the types of areas measured in comparison with previous tools. In the end, only one item in the final measure addressed the topic of communication, which is represented in multiple items on the CAHPS and the MHFI, because communication items clustered entirely at the easiest levels in the estimate of item-difficulty analysis. In contrast, four items related to decision-making interactions with providers and three items related to cultural and linguistic competence in care, areas missing on the CAHPS and the MHFI, were at higher levels of difficulty. Thus those concepts missing on the other measures are actually among the items that best discriminate high levels of family-centered care from lower levels.

### Limitations of the Study

The convenience sample recruited through state family organizations is not fully representative of families with CYSHCN as identified in the 2009–2010 NS-CSHCN. Families of color are underrepresented. Children of family members responding had higher needs and more limitations than are represented in the NS-CSHCN study population. Respondents also had many years of experience with the health care system for their children. Therefore, there needs to be confirmation that these findings apply to a more representative sample. At the same time, the length of experience and the level of service needs of the children suggest that respondents were particularly able to reflect on the experiences of family-centered care.

### Future Research

Future studies with this measure need to address representativeness of the sample. Additionally, next steps include validating the tool against other measures related to family-centered care, assessing test–retest reliability, and testing the tool’s ability to reflect changes in families’ perceptions of care after interventions to improve family-centered care. To address the growing diversity within the country, studies to develop versions of the measure in languages other than English need to be undertaken.

## Conclusion

This project yielded a robust and psychometrically sound instrument for which there has been substantial interest from health care providers, researchers, and family groups. The FCCA provides an important alternative to existing measures of families’ experiences of health care, including the CAHPS, which does not address the full range of topical areas deemed important to families, and the MHFI, which lacks the rigorous psychometric evaluation of the FCCA. The findings from this study also provide compelling evidence of the value of families taking substantive roles as researchers in the development of quality measures. The high level of validity of the family-created questions in this study indicates how consistent the concepts in the developed questions are with the expectations of families across all demographic groups. This model of partnership in research, with consumers themselves in the lead, provides an important model for future quality measure development.
